# 2324 A community-academic partnership to understand the correlates of successful aging in place (year 2)

**DOI:** 10.1017/cts.2018.236

**Published:** 2018-11-21

**Authors:** Kimberly Vasquez, Dozene Guishard, William Dionne, Alexandra Jurenko, Caroline Jiang, Cameron Coffran, Andrea Ronning, Glenis George-Alexander, Cynthia Mofunanya, Onassis C. Ceballo, Lisa Tsatsas, Barry S. Coller, Jonathan N. Tobin, Rhonda G. Kost

**Affiliations:** 1 Rockefeller University; 2 Clinical Directors Network

## Abstract

OBJECTIVES/SPECIFIC AIMS: Objective: The Rockefeller University Center for Clinical and Translational Science (RU-CCTS), Clinical Directors Network (CDN), and Carter Burden Network (CBN), a multisite senior services organization serving East Harlem, NY, formed a community-academic partnership to examine the use of a simple validated surrogate measure of overall health status and frailty in this population. Many CBN seniors are racial/ethnic minorities, low-income, and suffer from multiple chronic conditions, depression and food insecurity. Multiple biological, musculoskeletal, psychosocial and nutritional factors contribute to frailty, which has been defined variously in senior health outcomes research. The CTSA-funded Pilot Project aims to: (1) Engage CBN seniors and stakeholders in priority-setting, joint protocol development, research conduct, analysis, and dissemination; (2) Characterize the health status of the CBN seniors using validated measures; (3) Establish database infrastructure for current and future research; (4) Understand how health and senior activities information can be used to create programs to improve senior health. METHODS/STUDY POPULATION: Methods: (1) CEnR-Navigation, a collaborative program/process that consists of semistructured meetings and activities facilitated by expert Navigators, was used for partnership development and to engage Carter Burden seniors to refine priorities and research questions, provide feedback on study design and conduct, and analyze and disseminate results. (2) Standard physical measurements and validated survey instruments were used to collect health information; target enrollment is 240 seniors across 2 sites (1 hosted within a subsidized housing facility and Social Model Adult Day Program). (3) A REDCap-based platform was designed for data capture and import. Individual attendance at senior activities for the prior year was extracted from existing records. The primary outcome is frailty, as measured by validated walk/balance tests (Short Physical Performance Battery). Secondary outcomes include measures of engagement, and association of use of services/activities with the primary outcome. RESULTS/ANTICIPATED RESULTS: (1) In total, 29 residents and 14 other stakeholders engaged in partnership-building, study design and implementation. (2) From May to November 2017, 98 participants were enrolled from site 1 (a residential site). Enrollment at site 2 (a senior center), begun in November, is projected for February completion. Characteristics of site 1 participants: median age=63.6 years; Hispanic, 44.90% (44); White, 13.89% (10), Black, 62.50% (45); Asian, 4.17% (3); American Indian or Alaskan Native, 2.78% (2), and Other, 16.67% (12). Educational attainment: 51.04% (49) had not completed high school; 19.79% (19) were high school graduates; 18.75% (18) completed some college, and 10.42% (10) were college graduates. For the 85 participants reporting annual income: 64.71% (55) reported <$10,000; 28.24% (24) reported $10,000–$15,000; 7.06% (6) were among the ranges from $15,000 to $50,000. The average body mass index (BMI) was 30, which is obese. For 83.67% (82) of site 1 participants, the BMI was in the range of overweight or obese. Half of participants (49) reported health literacy barriers in the Single Item Health Literacy Survey. Demographics and Frailty assessments (walk and balance tests) for participants enrolled at both sites will be reported. (3) Activity participation data for July 2016–November 2017 were recovered for 507 sessions at site 1 and are being analyzed. DISCUSSION/SIGNIFICANCE OF IMPACT: Here we report progress in developing a sustainable community-academic partnership, infrastructure and research capacity with the CBN senior services organization, and characterizing this at-risk population, of whom 71% have a high school education or less, 93% live in extreme poverty, and 84% are overweight or obese. A simple validated frailty measure in seniors will enable the acceleration of community-based translational research addressing senior health, and examine changes in this measure in relationship to the utilization of senior services.

